# Dynamic Behavior of Left Atrial Low‐Voltage Areas in Persistent Atrial Fibrillation: Implications for Substrate‐Guided Ablation

**DOI:** 10.1111/jce.70445

**Published:** 2026-07-08

**Authors:** Yunus Emre Özbebek, Özcan Özdemir, Sinan Boz, Haluk Furkan Şahan, Engin Algül, Sinan İşcen, Hamza Sunman, Tolga Han Efe

**Affiliations:** ^1^ Department of Cardiology Ankara Etlik City Hospital Ankara Türkiye; ^2^ Department of Cardiology University of Health Sciences Istanbul Türkiye

**Keywords:** atrial remodeling, electroanatomical mapping, isoproterenol, low‐voltage areas, persistent atrial fibrillation, voltage mapping dynamics

## Abstract

**Background:**

Low‐voltage areas (LVAs) identified by electroanatomical mapping are commonly used to characterize the atrial substrate in persistent atrial fibrillation (PeAF). However, voltage measurements may vary according to rhythm and physiological state, and LVAs should not be considered synonymous with histological scar or fibrosis.

**Objective:**

To evaluate rhythm‐ and physiological state‐dependent changes in left atrial LVAs during atrial fibrillation (AF), early sinus rhythm after cardioversion (SR1), and sinus rhythm under isoproterenol infusion (SR2).

**Methods:**

This retrospective observational study included 49 patients with persistent AF undergoing first‐time catheter ablation. High‐density left atrial voltage maps were acquired during AF, early sinus rhythm after electrical cardioversion (SR1), and sinus rhythm during isoproterenol infusion (SR2). Bipolar LVAs were defined as voltage amplitudes < 0.3 mV during AF and < 0.5 mV during sinus rhythm. Unipolar LVAs were defined using corresponding rhythm‐specific thresholds of < 0.71 mV during AF and < 0.83 mV during sinus rhythm, whereas dense bipolar LVAs were defined as bipolar voltage amplitudes < 0.2 mV. Global and regional LVA burden was quantified, and complete paired global analyses included 48 patients.

**Results:**

Global LVA burden decreased stepwise across mapping conditions. Bipolar LVA ratio decreased from 17.25% (IQR, 11.40–31.76) during AF to 8.70% (IQR, 6.98–16.17) during SR1 and 5.20% (IQR, 2.60–10.93) during SR2 (*p* < 0.001). Similar reductions were observed for unipolar and dense bipolar LVA ratios (both *p* < 0.001). Regional analysis showed significant heterogeneity in bipolar LVA reduction, whereas unipolar and dense bipolar changes were more homogeneous. Larger left atrial volume was associated with less pronounced bipolar and unipolar LVA reduction. AF duration category was not significantly associated with LVA reduction.

**Conclusion:**

Voltage‐defined LVAs in PeAF vary substantially according to rhythm and physiological state. These findings suggest that single‐condition voltage mapping may incompletely characterize the atrial substrate and should be interpreted as dynamic electroanatomical changes rather than evidence of fibrosis or scar regression.

## Graphical abstract

Patient selection and analysis flow diagram. The exact pre‐inclusion denominator and reliable reason‐specific exclusion counts could not be reconstructed because a prospectively maintained screening log was unavailable. The diagram summarizes the final study cohort and analysis‐specific sample sizes. AF, atrial fibrillation; LVA, low‐voltage area; SR1, early sinus rhythm after cardioversion; SR2, sinus rhythm during isoproterenol infusion.

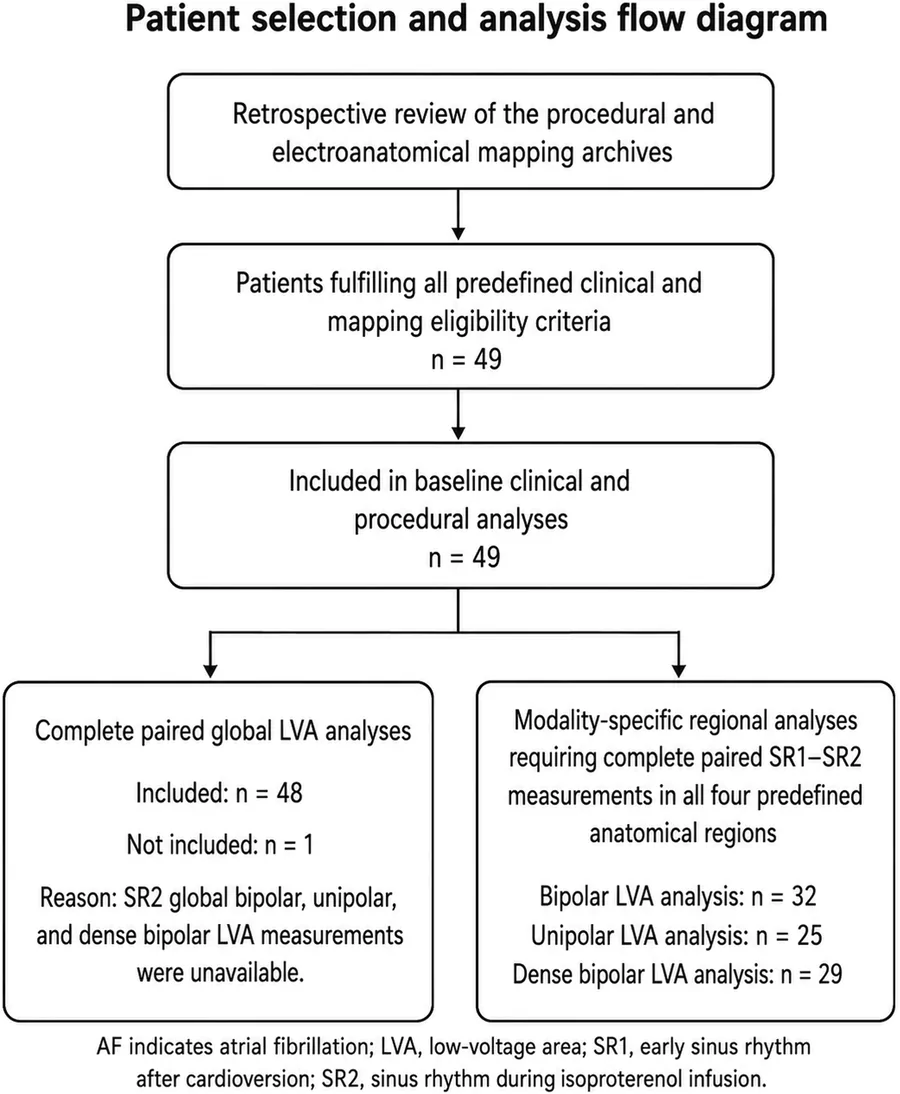

## 1 Introduction

Pulmonary vein isolation (PVI) remains the cornerstone of catheter ablation for atrial fibrillation (AF). However, outcomes after PVI alone are less consistent in patients with persistent AF (PeAF), in whom atrial remodeling and extrapulmonary vein substrate may contribute to arrhythmia maintenance. Low‐voltage areas (LVAs) identified by electroanatomical mapping have therefore been proposed as markers of abnormal atrial substrate and as potential targets for adjunctive ablation. Although the presence and extent of LVAs have been associated with atrial arrhythmia recurrence, the clinical benefit of LVA‐guided ablation remains inconsistent across studies [1, 2].

An important limitation of voltage‐guided substrate assessment is that measured atrial voltage is influenced not only by underlying tissue characteristics but also by rhythm, activation rate, wavefront direction, catheter orientation, tissue contact, and mapping methodology. Previous studies have demonstrated substantial differences between voltage maps obtained during AF and sinus rhythm, indicating that maps acquired under different rhythm conditions are not directly interchangeable [3]. Consequently, a voltage map obtained under a single rhythm or physiological condition may incompletely characterize voltage‐defined atrial abnormalities.

In patients presenting in PeAF, substrate mapping is frequently performed shortly after electrical cardioversion. However, the early post‐cardioversion period may be affected by atrial stunning and transient electrophysiological changes related to the preceding arrhythmia [4, 5]. Pharmacological adrenergic stimulation with isoproterenol can modify sinus rate, atrial activation, and atrial mechanical function and has been reported to improve left atrial appendage function during the early post‐cardioversion period [6]. Nevertheless, it remains uncertain whether voltage‐defined LVAs identified immediately after cardioversion remain stable during isoproterenol infusion. Previous investigations have mainly compared voltage mapping during AF with mapping during sinus rhythm, whereas the additional changes occurring between early post‐cardioversion sinus rhythm and sinus rhythm during adrenergic stimulation remain insufficiently characterized.

Clarifying this variability may be clinically relevant because substrate‐guided ablation decisions are commonly based on a single voltage map. Regions classified as LVAs under only one mapping condition may not represent stable voltage abnormalities, whereas regions that remain low‐voltage across different rhythm and physiological conditions may provide distinct substrate information. Dynamic voltage assessment may therefore provide complementary information for interpreting potential substrate targets, although whether this approach improves procedural or clinical outcomes remains unknown.

Accordingly, the present study compared global and regional bipolar, unipolar, and dense bipolar LVA burden under three sequential mapping conditions: ongoing AF, early sinus rhythm after electrical cardioversion (SR1), and sinus rhythm during isoproterenol infusion (SR2). We hypothesized that voltage‐defined LVA burden would vary across these conditions and that the magnitude and regional distribution of change would differ according to mapping modality. The study was designed to characterize these dynamic electroanatomical changes and was not intended to determine the clinical efficacy of dynamic LVA‐guided ablation.

## 2 Materials and Methods

### 2.1 Study Design and Population

This retrospective observational study included adult patients with symptomatic persistent atrial fibrillation (PeAF) who underwent first‐time catheter ablation at our institution. Persistent AF was defined as continuous AF lasting from 1 week to 1 year, and long‐standing persistent AF was defined as continuous AF lasting more than 1 year. Clinical, echocardiographic, laboratory, procedural, and electroanatomical mapping data were obtained from institutional electronic medical records and procedural archives.

Patients were eligible for inclusion if they had high‐density left atrial voltage maps acquired under all three predefined mapping conditions: during AF, during early sinus rhythm after electrical cardioversion (SR1), and during sinus rhythm under isoproterenol infusion (SR2). Only maps with at least 1000 accepted mapping points were included. Patients in whom electrical cardioversion failed to restore sinus rhythm before SR mapping were excluded.

The exclusion criteria were as follows: left atrial diameter > 55 mm on echocardiography within the preceding year, acute coronary syndrome within the previous 12 weeks, severe aortic or mitral valvular disease, adult congenital heart disease, previous percutaneous or surgical AF ablation, previous atrioventricular junction ablation, previous cardiac surgery involving the left or right atrium, New York Heart Association class IV heart failure symptoms, medical conditions likely to limit survival to less than 1 year, contraindication to oral anticoagulation, AF attributed to a reversible condition, pregnancy, and inability to provide informed consent at the time of the index procedure.

Because the study was based on retrospective procedural and electroanatomical mapping archives rather than a prospectively maintained screening log, the exact number of patients assessed before application of the eligibility criteria and reliable reason‐specific pre‐inclusion exclusion counts could not be reconstructed. Accordingly, no estimated screening denominator or exclusion counts were reported. Patients were not excluded solely because of the absence of LVAs.

A total of 49 patients fulfilled the predefined clinical and mapping eligibility criteria and were included in the study cohort and baseline analyses. Complete paired global LVA measurements across AF, SR1, and SR2 were available for 48 patients. One patient was excluded only from the paired global analyses because SR2 global bipolar, unipolar, and dense bipolar LVA measurements were unavailable. Complete paired measurements were also required separately for each modality‐specific regional analysis. The patient selection and analysis process is summarized in the accompanying flow diagram.

### 2.2 Ethical Approval

The study was conducted in accordance with the principles of the Declaration of Helsinki and was approved by the local institutional ethics committee (approval number/date: AEŞH‐BADEK2‐2026‐269/24.03.2026). Because of the retrospective design of the study, the requirement for written informed consent for study participation was waived.

### 2.3 Voltage Mapping Protocol

All procedures were performed using a three‐dimensional electroanatomical mapping system (CARTO 3, Biosense Webster, Inc.). After transseptal puncture, an anatomical map of the left atrium was created using a multipolar mapping catheter with five splines, each containing four 1 mm electrodes with 2–6–2 mm interelectrode spacing (PentaRay, Biosense Webster Inc.).

Voltage mapping was performed sequentially under three mapping conditions. First, a high‐density voltage map was acquired during ongoing AF. Electrical cardioversion was then performed, and a second voltage map was acquired during the early sinus rhythm after cardioversion (SR1). In routine practice, SR1 mapping was initiated as soon as a stable sinus rhythm and hemodynamic stability were achieved after cardioversion. Because this was a retrospective study, the exact cardioversion‐to‐SR1 mapping interval was not systematically recorded for all patients. A third voltage map was acquired during sinus rhythm under isoproterenol infusion (SR2), approximately 10 min after initiation of the infusion.

During sinus rhythm maps, coronary sinus pacing was performed at a cycle length of 500 ms according to the institutional mapping protocol. Pacing was generally performed from the distal coronary sinus to provide a consistent left atrial activation wavefront. During isoproterenol infusion, if the intrinsic sinus rate exceeded the paced cycle length, mapping was performed during the prevailing intrinsic sinus rhythm, and this was considered a potential source of cycle length‐related variability.

### 2.4 Isoproterenol Protocol

Isoproterenol was administered after completion of the SR1 map. The infusion was initiated at 2 μg/min and gradually titrated up to a maximum of 10 μg/min according to the sinus rate, blood pressure, and rhythm response. No predefined absolute heart‐rate target, percentage increase from baseline, or fixed duration of rate stability was specified in the institutional protocol. Dose escalation was discontinued when an adequate physiological response was considered to have been achieved or if hypotension, clinically significant symptoms, or sustained atrial tachyarrhythmia occurred.

The SR2 map was acquired approximately 10 min after initiation of the infusion, once the isoproterenol dose had been stabilized and no further dose escalation was planned during map acquisition. Coronary sinus pacing at a cycle length of 500 ms was continued when the intrinsic sinus rate remained slower than the pacing rate. If the intrinsic sinus rate exceeded the paced rate of 120 beats/min, mapping was performed during the prevailing intrinsic sinus rhythm. Exact heart‐rate and cycle‐length data at the time of SR2 mapping were not systematically recorded for all patients.

### 2.5 Point Acquisition and Electrogram Annotation

The CONFIDENSE module (Biosense Webster Inc.) was used for automated point acquisition. The predefined point acceptance criteria included position stability of 4 mm, local activation time stability of 4 ms, and cycle length filtering of 2%–5% when applicable. Because AF is an irregular rhythm with continuously varying electrogram morphology and activation sequence, AF maps were reviewed manually to ensure adequate point quality and tissue contact. During AF mapping, automated criteria were used as supportive acquisition filters rather than as strict indicators of organized activation. Electrograms were manually reviewed, and points with inadequate catheter contact, far‐field signals, noise, or visually inappropriate annotations were excluded.

The window of interest was adjusted for each mapping condition to include the relevant atrial electrograms and to exclude ventricular far‐field components. During AF, local bipolar voltage amplitude was annotated using the peak‐to‐peak amplitude of accepted atrial electrograms. During sinus rhythm and coronary sinus pacing, local activation timing and voltage annotation were confirmed using the same mapping system settings and manual review. Point acceptance criteria were otherwise kept consistent across mapping conditions as far as technically feasible.

### 2.6 Definition and Measurement of LVAs

LVAs were defined using predefined voltage thresholds. Bipolar LVAs were defined as areas with bipolar voltage amplitude < 0.3 mV during AF and < 0.5 mV during sinus rhythm. A bipolar threshold around 0.5 mV is commonly used to define atrial low‐voltage substrate during sinus rhythm, whereas lower AF‐specific thresholds have been proposed because voltage amplitude is rhythm‐dependent. Unipolar LVAs were defined using rhythm‐specific thresholds of < 0.71 mV during AF and < 0.83 mV during sinus rhythm. Dense bipolar LVAs were defined as areas with bipolar voltage amplitude < 0.2 mV. Dense bipolar LVA analysis was performed separately to evaluate regions with very low bipolar voltage amplitude, as prior mapping studies suggest that very low‐voltage tissue may behave differently from intermediate low‐voltage tissue across rhythm states.

The LVA area was measured using the “area measurement” tool within the CARTO system. Areas meeting the predefined color‐coded voltage thresholds were manually delineated on the electroanatomical map. Total LVA area and LVA ratio were calculated for each mapping condition. LVA ratio was defined as the percentage of the left atrial surface area occupied by LVAs. When available, both absolute LVA area and LVA ratio were analyzed.

If an LVA was detected using the multipolar mapping catheter, the region and its border zone were confirmed with a contact‐force sensing ablation catheter (SmartTouch, Biosense Webster Inc.) to ensure adequate tissue contact and to minimize false low‐voltage annotation.

### 2.7 Regional Segmentation

For regional analyses, the left atrium was divided into four anatomical regions: anterior wall, posterior wall, roof, and inferior wall. The anterior wall was defined as the region between the mitral annulus and the left atrial roof anterior to the pulmonary veins. The posterior wall was defined as the region between the pulmonary vein antra, extending from the roof superiorly to the inferior posterior left atrium. The roof was defined as the superior left atrial region connecting the superior pulmonary veins. The inferior wall was defined as the region between the inferior pulmonary veins and the mitral annulus. Regional LVA burden was calculated as the percentage of each predefined anatomical region occupied by LVAs.

### 2.8 Blinding and Reproducibility of LVA Measurement

All LVA measurements were performed by two observers experienced in electroanatomical mapping. Each observer independently measured LVA areas using the same predefined voltage thresholds and anatomical segmentation. Measurements were performed using deidentified procedural maps. Discrepancies between observers were resolved by consensus. When disagreement persisted, the final measurement was adjudicated by a senior electrophysiologist. Because this was a retrospective study, formal intraobserver and interobserver reproducibility analyses were not available.

### 2.9 Statistical Analysis

Statistical analyses were performed using SPSS software version 29.0 (IBM Corp., Armonk, NY, USA). Continuous variables were assessed for distribution using visual inspection of histograms and Q–Q plots. Normally distributed variables are presented as mean ± standard deviation, whereas non‐normally distributed variables are presented as median and interquartile range. Categorical variables are presented as counts and percentages.

Global LVA burden across AF, SR1, and SR2 was compared using the Friedman test. When the overall Friedman test was significant, pairwise comparisons were performed using Wilcoxon signed‐rank tests with Bonferroni correction. Regional SR1–SR2 LVA percentage changes were compared across anatomical regions using the Friedman test. Kendall's coefficient of concordance was used to estimate the magnitude of regional differences where appropriate.

Associations between LVA reduction and continuous clinical or echocardiographic variables were evaluated using Spearman's rank correlation analysis. Differences in LVA reduction according to categorical variables, including AF duration category, were assessed using the Mann–Whitney *U* test. Because these analyses were exploratory, they were interpreted cautiously. A two‐sided *p* < 0.05 was considered statistically significant.

## 3 Results

### 3.1 Baseline Clinical and Procedural Characteristics

Baseline clinical, echocardiographic, and procedural characteristics of the study population are summarized in Table 1. A total of 49 patients were included in the analysis. The mean age was 68.1 ± 6.4 years, and 24 patients (49.0%) were male. Hypertension was present in 43 patients (87.8%), whereas diabetes mellitus and coronary artery disease were present in 6 (12.2%) and 7 (14.3%) patients, respectively. AF duration was > 12 months in 17 patients (34.7%). The mean left atrial diameter was 49.0 ± 4.3 mm, and the mean left ventricular ejection fraction was 49.0 ± 10.7%. B‐type natriuretic peptide levels showed a markedly skewed distribution, with a median value of 367 pg/mL (IQR, 258–1319 pg/mL).

**Table 1:** Baseline clinical, echocardiographic, and laboratory characteristics of the study population (*n* = 49).

Variable	Value
Age, years	68.1 ± 6.4
Male sex, *n* (%)	24 (49.0)
Hypertension, *n* (%)	43 (87.8)
Diabetes mellitus, *n* (%)	6 (12.2)
Coronary artery disease, *n* (%)	7 (14.3)
Atrial fibrillation duration > 12 months, *n* (%)	17 (34.7)
Left atrial diameter, mm	49.0 ± 4.3
Left ventricular ejection fraction, %	49.0 ± 10.7
Systolic pulmonary artery pressure, mmHg	37.9 ± 5.9
Estimated glomerular filtration rate, mL/min	64.5 ± 12.6
B‐type natriuretic peptide, pg/mL, median (IQR)	367 (258–1319)
Number of AF mapping points	3113 ± 683

*Note:* Data are presented as mean ± standard deviation, median (interquartile range), or *n* (%), as appropriate.

### 3.2 Mapping Density

Mapping density was high across all three mapping conditions. The median number of accepted mapping points was 3048 (IQR, 2589–3744; range, 1917–4620) during AF, 3014 (IQR, 2528–3493; range, 1841–4842) during SR1, and 3161 (IQR, 2660–3596; range, 1735–4324) during SR2. All included maps exceeded the predefined minimum of 1000 accepted mapping points.

### 3.3 Global Changes in LVA Burden Across Mapping Conditions

Complete paired global LVA measurements across AF, SR1, and SR2 were available for 48 patients; SR2 global bipolar, unipolar, and dense bipolar LVA measurements were unavailable for one patient.

Global LVA burden showed a stepwise reduction across the three mapping conditions (Table 2). Bipolar LVA ratio was highest during AF and progressively decreased after restoration of sinus rhythm and after isoproterenol infusion: 17.25% (IQR, 11.40–31.76) during AF, 8.70% (IQR, 6.98–16.17) during SR1, and 5.20% (IQR, 2.60–10.93) during SR2 (Friedman *χ* ^2^ = 90.931, df = 2, *n* = 48, *p* < 0.001). Pairwise comparisons demonstrated significant reductions from AF to SR1, from SR1 to SR2, and from AF to SR2 after Bonferroni correction (all *p* < 0.001).

**Table 2:** Global LVA burden across mapping conditions.

Mapping modality	*n*	AF, median (IQR)	SR1, median (IQR)	SR2, median (IQR)	Friedman *χ* ^2^	*p* value	Pairwise comparisons
Bipolar LVA ratio, %	48	17.25 (11.40–31.76)	8.70 (6.98–16.17)	5.20 (2.60–10.93)	90.931	< 0.001	AF > SR1, SR1 > SR2, AF > SR2; all *p* < 0.001
Unipolar LVA ratio, %	48	8.67 (5.06–21.71)	3.70 (1.77–5.36)	3.00 (0.00–3.99)	80.962	< 0.001	AF > SR1, SR1 > SR2, AF > SR2; all *p* < 0.001
Dense bipolar LVA ratio, %	48	10.20 (7.40–15.30)	5.90 (3.20–8.65)	3.28 (1.01–6.63)	95.032	< 0.001	AF > SR1, SR1 > SR2, AF > SR2; all *p* < 0.001

*Note:* Data are presented as median (interquartile range). Global LVA burden was compared across mapping conditions using the Friedman test. Pairwise comparisons were performed using Wilcoxon signed‐rank tests with Bonferroni correction.

Abbreviations: AF, atrial fibrillation; LVA, low‐voltage area; SR1, early sinus rhythm after cardioversion; SR2, sinus rhythm after isoproterenol infusion.

Similar stepwise reductions were observed for unipolar and dense bipolar LVA ratios. Unipolar LVA ratio decreased from 8.67% (IQR, 5.06–21.71) during AF to 3.70% (IQR, 1.77–5.36) during SR1 and 3.00% (IQR, 0.00–3.99) during SR2 (Friedman *χ* ^2^ = 80.962, df = 2, *n* = 48, *p* < 0.001). Dense bipolar LVA ratio decreased from 10.20% (IQR, 7.40–15.30) during AF to 5.90% (IQR, 3.20–8.65) during SR1 and 3.28% (IQR, 1.01–6.63) during SR2 (Friedman *χ* ^2^ = 95.032, df = 2, *n* = 48, *p* < 0.001). Pairwise comparisons were significant for all comparisons in both modalities after Bonferroni correction (all *p* < 0.001).

Absolute LVA area showed a comparable pattern and is presented in Table S1. Bipolar LVA area decreased from 43.30 cm^2^ (IQR, 26.30–82.00) during AF to 22.00 cm^2^ (IQR, 10.78–41.10) during SR1 and 12.20 cm^2^ (IQR, 5.65–22.00) during SR2 (Friedman *χ* ^2^ = 94.128, df = 2, *n* = 48, *p* < 0.001). Unipolar and dense bipolar LVA areas also decreased significantly across AF, SR1, and SR2 (both *p* < 0.001), with all pairwise comparisons remaining significant after Bonferroni correction.

### 3.4 Regional LVA Changes From SR1 to SR2

**Figure 1:**
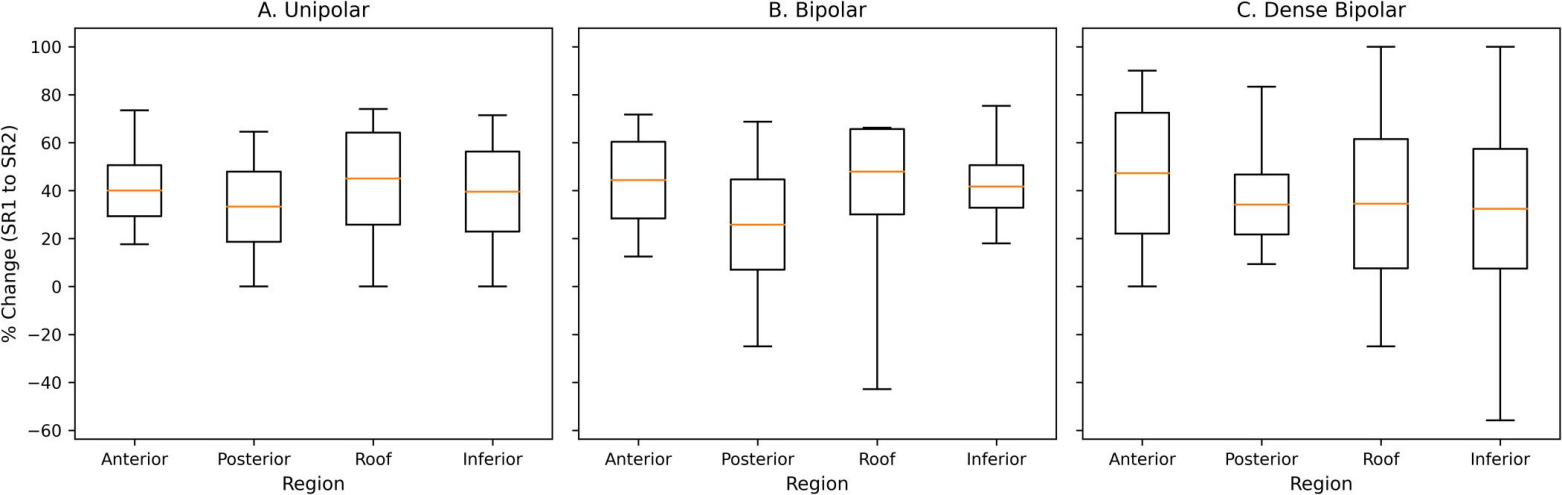
Regional percentage change in LVA burden from SR1 to SR2 according to mapping modality. Positive values indicate a reduction in LVA burden from SR1 to SR2, whereas negative values indicate an increase. Panel (A) shows regional percentage changes in unipolar LVA burden, with no significant difference among the anterior, posterior, roof, and inferior regions (Friedman test, *p* = 0.070). Panel (B) shows regional percentage changes in bipolar LVA burden, demonstrating significant regional heterogeneity, with the posterior region showing the smallest median reduction (Friedman test, *p* < 0.001). Panel (C) shows regional percentage changes in dense bipolar LVA burden, with no significant regional differences (Friedman test, *p* = 0.633). Boxes represent the median and interquartile range, and whiskers indicate the minimum and maximum values.

Regional percentage reductions in LVA burden from SR1 to SR2 according to mapping modality are presented in Table 3 and illustrated in Figure 1. In bipolar voltage mapping, a significant regional difference in LVA reduction was observed across atrial segments (Friedman *χ* ^2^ = 18.911, df = 3, *p* < 0.001). The greatest relative reductions were observed in the roof and anterior walls, whereas the posterior wall demonstrated the smallest percentage change.

**Table 3:** Regional percentage reduction in LVA burden from SR1 to SR2 according to mapping modality.

Mapping modality	*n*	Anterior wall, median (IQR)	Posterior wall, median (IQR)	Roof, median (IQR)	Inferior wall, median (IQR)	Friedman *χ* ^2^ (df = 3)	*p* value
Unipolar	25	40.0 (21.32)	33.3 (29.32)	45.0 (38.33)	39.6 (33.41)	7.049	0.070
Bipolar	32	44.4 (31.96)	25.8 (37.66)	47.9 (35.61)	41.7 (17.75)	18.911	< 0.001
Dense bipolar	29	47.3 (50.37)	34.2 (25.01)	34.5 (53.90)	32.4 (49.92)	1.716	0.633

*Note:* Data are presented as median (interquartile range). Positive values indicate a reduction in LVA burden from SR1 to SR2, whereas negative values indicate an increase. Regional comparisons were performed using the Friedman test. The sample size differs among mapping modalities because regional analyses included only patients with complete SR1–SR2 paired measurements for all four predefined anatomical regions within the corresponding mapping modality.

Abbreviations: LVA, low‐voltage area; SR1, early sinus rhythm after cardioversion; SR2, sinus rhythm after isoproterenol infusion.

**Figure 2:**
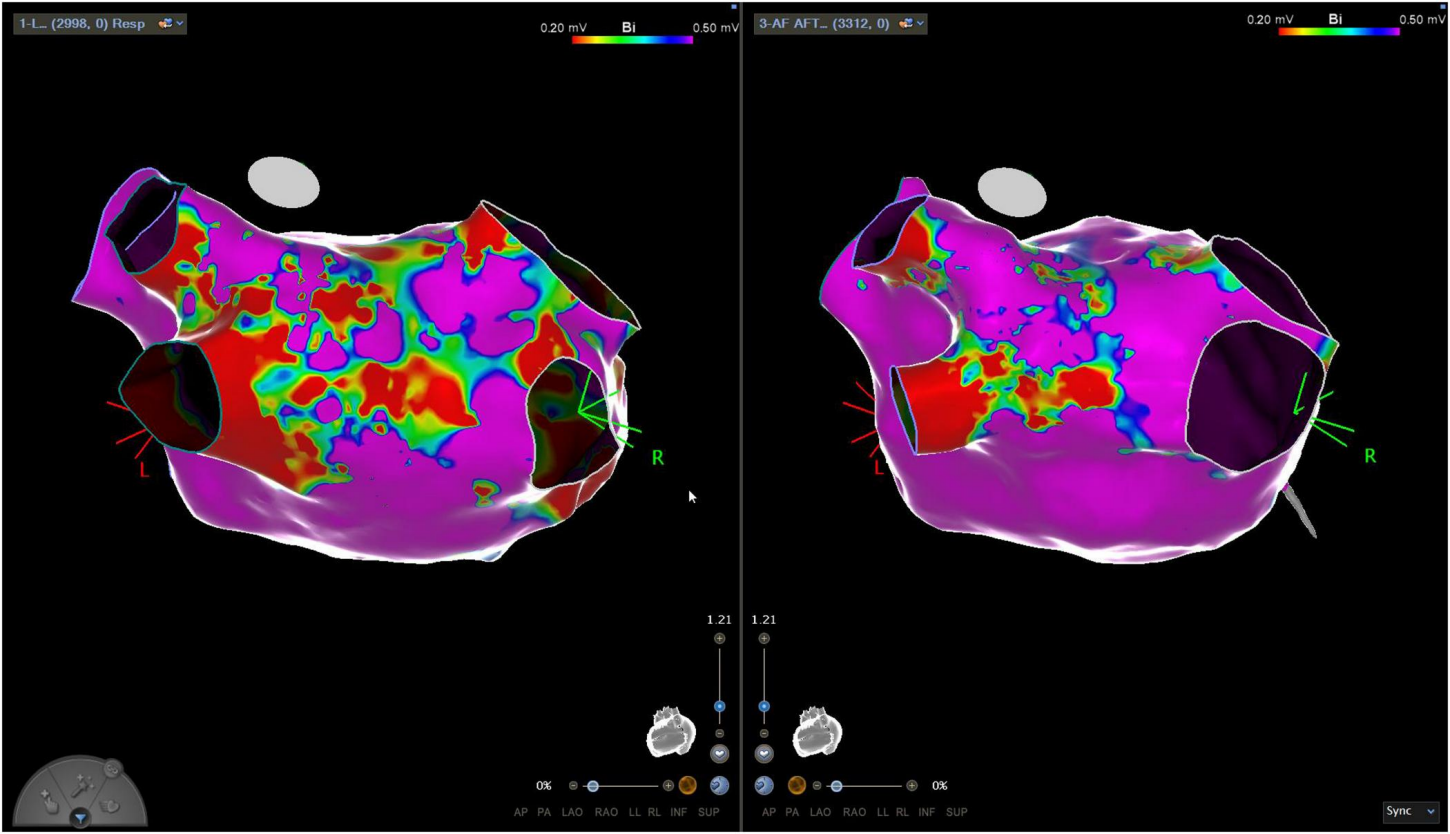
Representative bipolar voltage maps. Representative left atrial bipolar voltage maps obtained in the same patient during early sinus rhythm after cardioversion (SR1, left panel) and during sinus rhythm under isoproterenol infusion (SR2, right panel). The maps demonstrate a reduction in bipolar LVA burden from SR1 to SR2.

**Figure 3:**
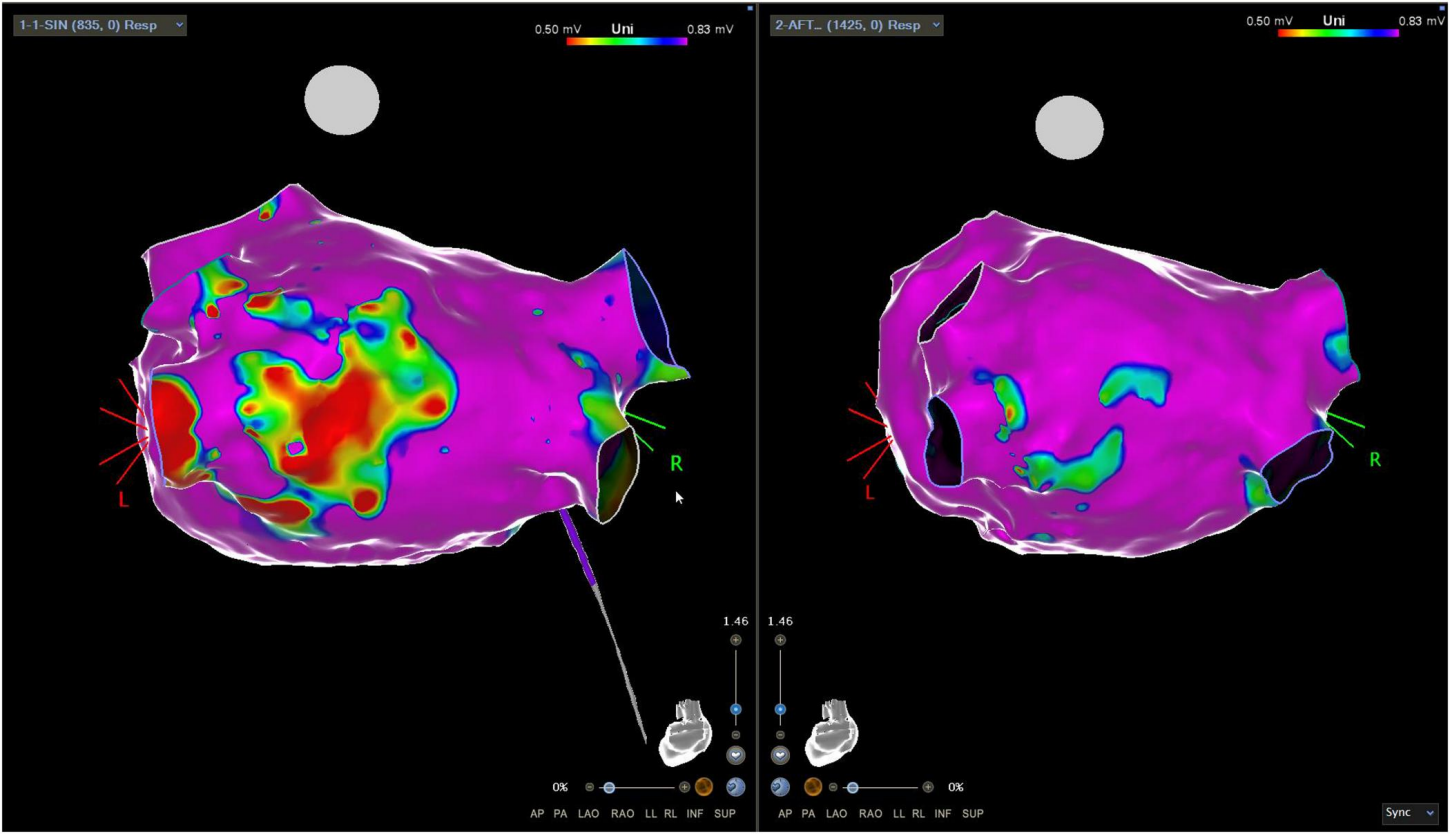
Representative unipolar voltage maps. Representative left atrial unipolar voltage maps obtained in the same patient during early sinus rhythm after cardioversion (SR1, left panel) and during sinus rhythm under isoproterenol infusion (SR2, right panel). Unipolar LVAs were defined as areas with voltage amplitudes < 0.83 mV during sinus rhythm. The maps demonstrate a reduction in unipolar LVA burden from SR1 to SR2.

In contrast, no statistically significant regional differences were detected in unipolar mapping (*χ* ^2^ = 7.049, df = 3, *p* = 0.070) or dense bipolar mapping (*χ* ^2^ = 1.716, df = 3, *p* = 0.633), indicating a more homogeneous distribution of SR1–SR2 LVA reduction across atrial regions when assessed with these modalities (Figures 2 and 3).

### 3.5 Association Between Left Atrial Size and LVA Reduction

In exploratory correlation analyses, a larger left atrial size was associated with less pronounced LVA reduction from SR1 to SR2. Left atrial volume measured during AF correlated inversely with bipolar LVA percentage reduction (Spearman's *ρ* = −0.439, *p* = 0.002) and unipolar LVA percentage reduction (*ρ* = −0.602, *p* < 0.001). Left atrial diameter showed similar inverse associations with bipolar (*ρ* = −0.413, *p* = 0.004) and unipolar LVA percentage reduction (*ρ* = −0.434, *p* = 0.005). No significant correlation was observed between left atrial volume and dense bipolar LVA percentage reduction (*ρ* = 0.049, *p* = 0.746).

### 3.6 Association Between AF Duration and LVA Reduction

AF duration category was not significantly associated with SR1–SR2 LVA reduction. Bipolar LVA percentage reduction did not differ significantly between patients with AF duration ≤ 12 months and those with AF duration > 12 months (Mann–Whitney *U* = 190.0, *p* = 0.150). Similarly, no significant differences were observed for unipolar LVA percentage reduction (*U* = 136.0, *p* = 0.070) or dense bipolar LVA percentage reduction (*U* = 235.0, *p* = 0.793).

## 4 Discussion

Our principal findings can be summarized as follows. First, substantial differences in both bipolar and unipolar LVAs were observed between AF and sinus rhythm, confirming the rhythm‐dependent nature of voltage mapping. Importantly, a considerable proportion of voltage‐defined LVA appeared to be rhythm‐dependent, suggesting that not all LVAs necessarily represent fixed structural disease. These findings indicate that voltage‐defined LVAs should not be interpreted uniformly as fixed structural disease, because their extent may vary according to rhythm and physiological state. Second, even after restoration of sinus rhythm and subsequent isoproterenol infusion, significant regional heterogeneity in bipolar LVA reduction persisted, with the smallest percentage reduction observed in the posterior wall. Nevertheless, a marked reduction in both bipolar and unipolar LVA areas was observed following isoproterenol infusion, predominantly outside dense bipolar LVA regions. These findings support the concept that a relevant proportion of voltage‐defined LVAs may reflect functional electroanatomical properties rather than exclusively fixed structural remodeling. Third, larger left atrial volume was associated with less pronounced LVA reduction, whereas AF duration category was not significantly associated with LVA reduction. The inverse relationship between left atrial volume and voltage recovery suggests that advanced structural remodeling may limit the reversibility of voltage abnormalities, linking atrial size not only to LVA burden but also to substrate plasticity. Approximately 30%–50% of patients with PeAF are believed to harbor arrhythmogenic substrate beyond the pulmonary veins. In line with this, the presence and extent of left atrial LVAs have consistently been associated with higher recurrence rates following PVI alone [7, 8]. However, inconsistent outcomes of LVA ablation strategies may be explained by several factors [9, 10]. These observations may help explain why LVA‐guided ablation strategies have yielded heterogeneous results. However, the present study was not designed to determine whether dynamic LVA assessment improves ablation outcomes.

Several factors likely contribute to variability in LVA interpretation. First, voltage cut‐off values used to define LVAs vary widely across studies, and the commonly applied bipolar threshold of 0.5 mV may not optimally discriminate pathological atrial tissue across different mapping conditions and clinical contexts [8, 11]. The absence of standardized voltage cut‐off values remains a major limitation of voltage‐guided strategies; this variability likely reflects both biological heterogeneity in atrial remodeling and methodological differences across mapping approaches.

Second, there is no consensus regarding the optimal rhythm for substrate mapping. Mapping during AF consistently yields larger LVAs compared with sinus rhythm, likely reflecting rhythm‐dependent conduction slowing and wavefront disorganization; thus, AF mapping may unmask functional and dynamic substrates that remain undetected during sinus rhythm and should be considered complementary rather than interchangeable with SR mapping [3, 12].

Third, bipolar electrogram amplitudes are influenced by multiple non‐fibrotic factors, including atrial wall thickness, electrode size and interelectrode spacing, catheter orientation, tissue contact, atrial activation rate, and wavefront directionality, all of which may affect voltage measurements independent of underlying fibrosis [8]. In contrast, unipolar voltage mapping is less dependent on electrode orientation and interelectrode spacing [13]. In the present study, unipolar mapping demonstrated changes parallel to those observed with bipolar mapping, supporting its complementary role in characterizing the atrial substrate.

From a clinical perspective, reliance on a single static voltage map may lead to both underestimation of critical substrate and overestimation of ablation targets, potentially resulting in unnecessary extensive ablation and iatrogenic atrial tachycardias without improving outcomes [9, 14]. Our findings support a shift toward physiology‐based, individualized substrate assessment integrating mapping across different functional states. Such an approach may improve substrate characterization and optimize ablation strategies in persistent AF.

Finally, the strong association between left atrial volume and voltage recovery suggests that advanced remodeling limits substrate reversibility, supporting the concept of atrial cardiomyopathy as a dynamic and progressive process.

Although this study was not designed to evaluate clinical outcomes after ablation, our findings provide a mechanistic framework that may explain the heterogeneous results of LVA‐guided ablation strategies reported in previous trials. Future prospective studies integrating dynamic voltage mapping with clinical follow‐up are warranted to determine whether physiology‐guided substrate assessment improves procedural success and reduces arrhythmia recurrence.

### 4.1 Why This Study Matters

This study highlights that atrial substrate characterization based on a single static voltage map may provide an incomplete representation of the atrial substrate in patients with persistent AF. The findings emphasize that voltage‐defined LVAs are not necessarily static and may vary according to rhythm and physiological state. Therefore, dynamic voltage assessment may offer complementary information beyond conventional single‐condition mapping.

From a clinical perspective, these findings may help explain the inconsistent outcomes observed in LVA‐guided ablation studies. Reliance on static low‐voltage thresholds may result in both under‐recognition of relevant electroanatomical substrate and overestimation of potential ablation targets. However, the present study was not designed to determine whether dynamic LVA assessment improves ablation outcomes, and the clinical implications should therefore be interpreted cautiously.

By demonstrating the dynamic behavior of LVAs during AF, after cardioversion, and following pharmacological modulation with isoproterenol, this study supports further investigation of individualized, physiology‐based substrate assessment. Future studies should determine whether integrating rhythm‐dependent voltage mapping and functional substrate evaluation can improve patient selection, refine ablation targets, and enhance outcomes in PeAF.

### 4.2 Limitations

This study has several limitations. First, the sample size was relatively small and derived from a single center, which may limit the generalizability of the findings. Second, the observational and retrospective design precludes causal inference and may introduce selection bias, particularly because only patients with complete voltage maps during AF, SR1, and SR2 were included. Third, the exact interval between cardioversion and SR1 mapping was not systematically recorded for all patients, although SR1 mapping was performed after restoration of a stable sinus rhythm. Fourth, AF voltage mapping is inherently less reproducible than sinus rhythm mapping because electrogram morphology and activation sequence vary continuously during AF. Although all maps were manually reviewed for point quality and tissue contact, residual variability related to AF mapping cannot be excluded.

Fifth, sinus rhythm maps were obtained during coronary sinus pacing, which may introduce wavefront‐dependent voltage changes or pseudo‐low‐voltage regions in some left atrial segments. Sixth, the isoproterenol protocol did not include a predefined absolute heart‐rate target, percentage increase from baseline, or fixed duration of rate stability. Furthermore, the exact heart rate and cycle length during SR2 mapping were not systematically available. Therefore, part of the observed SR1–SR2 voltage change may have been related to differences in activation rate or cycle length.

Another important limitation is that rhythm‐specific voltage thresholds were used to define LVAs, namely < 0.3 mV for bipolar mapping during AF and < 0.5 mV during sinus rhythm, with corresponding rhythm‐specific unipolar thresholds. Therefore, the present study does not compare LVA burden using identical voltage cutoffs across all rhythm states. Although these predefined thresholds were selected based on previously proposed criteria, the magnitude of the observed differences between AF, SR1, and SR2 may partly reflect threshold selection rather than purely physiological changes in atrial substrate. Because identical‐threshold remapping was not available in the retrospective data set, we could not perform a sensitivity analysis using uniform bipolar cutoffs across AF, SR1, and SR2.

Finally, because neither histology nor cardiac magnetic resonance imaging was performed, LVAs should not be considered synonymous with fibrosis or histological scar in the present study. Future multicenter studies with larger populations, standardized mapping protocols, multimodality substrate validation, and longitudinal follow‐up are warranted to determine which dynamic LVA patterns are clinically relevant and whether they should be incorporated into substrate‐guided ablation strategies.

## 5 Conclusion

Voltage‐defined left atrial LVAs in PeAF vary substantially according to rhythm and physiological state. LVA burden decreased after restoration of sinus rhythm and further after isoproterenol infusion, while a larger left atrial size was associated with less pronounced LVA reduction. These findings suggest that single‐condition voltage mapping may provide an incomplete representation of the atrial substrate. Because no histological or cardiac magnetic resonance validation was performed, these changes should be interpreted as dynamic electroanatomical LVA changes rather than evidence of fibrosis or scar regression.

## Funding

The authors have nothing to report.

## Supporting information


**Table 1:** Absolute LVA area across mapping conditions.

## Data Availability

The data that support the findings of this study are available on request from the corresponding author. The data are not publicly available due to privacy or ethical restrictions.
