# Supplementary material for: Dynamic Behavior of Left Atrial Low‐Voltage Areas in Persistent Atrial Fibrillation: Implications for Substrate‐Guided Ablation
Source: J Cardiovasc Electrophysiol. 2026 Jul 8;37(9):2005–13. doi: 10.1111/jce.70445 (PMC13576552; doi:10.1111/jce.70445)
Supplement: Supplementary file 1 [file JCE-37-2005-s001.docx]

**Supplementary Table 1. Absolute LVA area across mapping conditions**

| **Mapping modality** | n | AF, median (IQR) | SR1, median (IQR) | SR2, median (IQR) | Friedman χ² | p value | Pairwise comparisons |
| --- | --- | --- | --- | --- | --- | --- | --- |
| Bipolar LVA area, cm² | 48 | 43.30 (26.30–82.00) | 22.00 (10.78–41.10) | 12.20 (5.65–22.00) | 94.128 | <0.001 | AF > SR1, SR1 > SR2, AF > SR2; all p < 0.001 |
| **Unipolar LVA area, cm²** | 48 | 22.40 (14.75–42.30) | 10.00 (3.50–14.00) | 8.00 (0.00–10.16) | 92.897 | <0.001 | AF > SR1, SR1 > SR2, AF > SR2; all p < 0.001 |
| **Dense bipolar LVA area, cm²** | 48 | 32.00 (16.00–38.98) | 15.10 (5.38–29.88) | 6.35 (1.84–14.10) | 95.032 | <0.001 | AF > SR1, SR1 > SR2, AF > SR2; all p < 0.001 |

Data are presented as median (interquartile range). Global LVA area was compared across mapping conditions using the Friedman test. Pairwise comparisons were performed using Wilcoxon signed-rank tests with Bonferroni correction. LVA indicates low-voltage area; AF, atrial fibrillation; SR1, early sinus rhythm after cardioversion; SR2, sinus rhythm after isoproterenol infusion.
